# Reliability of Stereotactic Radiofrequency Ablation (SRFA) for Malignant Liver Tumors: Novice versus Experienced Operators

**DOI:** 10.3390/biology12020175

**Published:** 2023-01-22

**Authors:** Peter Schullian, Gregor Laimer, Edward Johnston, Daniel Putzer, Gernot Eberle, Gerlig Widmann, Yannick Scharll, Reto Bale

**Affiliations:** 1 Department of Radiology, Section of Interventional Oncology—Microinvasive Therapy (SIP), Medical University of Innsbruck, Anichstr. 35, 6020 Innsbruck, Austria; 2 The Royal Marsden Hospital, 203 Fulham Road, Chelsea, London SW3 6JJ, UK

**Keywords:** radiofrequency ablation, stereotaxy, reliability, liver, tumor

## Abstract

**Simple Summary:**

The aim of this article was to compare results of a novice with those of experienced interventional radiologists (IRs) for stereotactic radiofrequency ablation (SRFA) in terms of safety, technical success, and local tumor control. SRFA is a minimally invasive, potentially curative treatment option for malignant liver tumors. After retrospective analysis of single center data (January 2011–December 2018), 39 ablation sessions performed by a novice IR were compared to the results of three more experienced IRs using propensity score matching. No significant differences were observed when comparing the results of more experienced IRs with those of the novice IR regarding the rates of major complications, primary technical efficacy, and local recurrence. However, the median planning/placement time was significantly shorter for the experienced IRs. SRFA is a safe, effective, and reliable treatment option for malignant liver tumors and favorable outcomes can be achieved even by inexperienced operators with minimal supervision.

**Abstract:**

Purpose: To compare the results of a novice with those of experienced interventional radiologists (IRs) for stereotactic radiofrequency ablation (SRFA) of malignant liver tumors in terms of safety, technical success, and local tumor control. Methods: A database, including all SRFA procedures performed in a single center between January 2011 and December 2018 was retrospectively analyzed. A total of 39 ablation sessions performed by a novice IR were compared to the results of three more experienced IRs. Comparative SRFA sessions were selected using propensity score matching considering tumor type, age, sex, tumor size, and tumor number as matching variables. Overall, 549 target tumors were treated in 273 sessions. Median tumor size was 2.2 cm (1.0–8.5 cm) for 178 hepatocellular carcinomas (HCCs) and 3.0 cm (0.5–13.0 cm) for 371 metastases. A median of 2 (1–11) tumors were treated per session. Results: No significant differences were observed when comparing the results of more experienced IRs with those of a novice IR regarding the rates of major complications (6.8% [16/234] vs. 5.1% [2/39]; *p* = 0.477), mortality (1.3% [2/234] vs. 0% [0/39]; *p* = 0.690), primary technical efficacy (98.5% [525/533] vs. 98.9% [94/95]; *p* = 0.735), and local recurrence (5.6% [30/533] vs. 5.3% [5/95]; *p* = 0.886). However, the median planning/placement time was significantly shorter for the experienced IRs (92 min vs. 119 min; *p* = 0.002). Conclusions: SRFA is a safe, effective, and reliable treatment option for malignant liver tumors and favorable outcomes can be achieved even by inexperienced operators with minimal supervision.

## 1. Introduction

Due to comparable overall survival rates, radiofrequency ablation (RFA) has gained increasing importance as an alternative treatment option to hepatic resection (HR) in the management of small hepatocellular carcinomas (HCCs) and colorectal liver metastasis (CRLMs) [1,2,3,4,5]. However, there are substantial differences in ablation practices between centers, mainly due to the lack of standardization and a wide range of technical possibilities. In addition, several studies [6,7,8] have shown a significant improvement in outcomes with increasing operator experience.

Stereotaxy translates pre-procedural plans defined on imaging to real patients using a Cartesian co-ordinate system [9]. Important methodological components such as trajectory planning and aiming device adjustment can be practiced before their use in clinical cases. In an earlier study, Widmann et al. [10] have already shown that there was no significant difference between an experienced and an inexperienced interventional radiologist (IR) in the outcome of SRFA for liver tumors.

The purpose of this study was to assess the safety, technical success, and local tumor control for SRFA of malignant liver tumors and to compare the results of a novice with those of more experienced IRs using propensity score matching.

## 2. Materials and Methods

### 2.1. Patient Selection

This retrospective, single-center study was approved by the institutional Review Board of Innsbruck (study number: AN4357) and written informed consent was obtained from all patients. All cases were reviewed in interdisciplinary tumor board meetings where treatment the plans were approved by consensus. 

**Figure 1:**
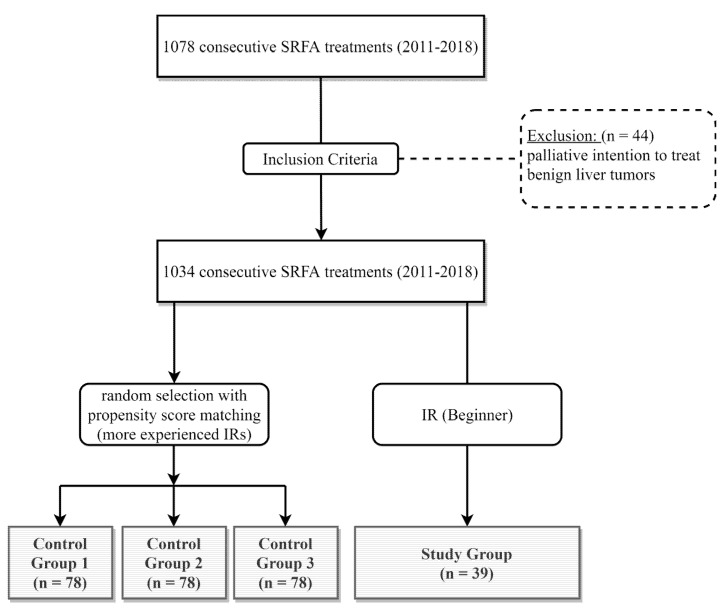
Flowchart of group assignment. IR = interventional radiologist.

A total of 1078 consecutive SRFA sessions were performed at our institution between January 2011 and December 2018. Forty-four ablation sessions in patients with portal venous invasion, extensive tumor spread with initial palliative intention to treat, and benign liver tumors were excluded from this analysis (Figure 1). The remaining 1034 ablations were subdivided according to the experience of the performing radiologist. Three interventional radiologists (IR1–3) were considered experienced (>2 years of SRFA experience) and one (IR4) was considered a novice. All consecutive ablations from IR4 (novice) were included in this analysis (n = 39). For comparison, 78 ablations from each of the other three more experienced radiologists (IR1–3) were selected by propensity score matching (R package “MatchIt” 1:2 matching) using the following matching variables: Tumor type, age, sex, tumor size, and tumor number. The baseline characteristics of these four groups are shown in Table 1.

Exclusion criteria for SRFA were a prothrombin activity <50%, platelet count of <50,000/mm^3^, and tumor location within 1 cm of the central bile duct. Tumor diagnosis was based on classic tumor enhancement patterns on multiphasic contrast MRI or CT scans and was confirmed by pre- or intraprocedural biopsy in all cases.

### 2.2. Patient Characteristics

A total of 237 Patients (76 women, 161 men) with a median age of 69 years (18–88) collectively underwent 273 liver SRFAs for treatment of 549 tumors. Histology characterized 150 (63.3%) HCCs, 9 (3.8) intrahepatic cholangiocarcinomas, and 78 (32.9%) metastatic tumors, with the majority (43/78, 55%) originating from colorectal cancer. Of those, 142/237 (59.9%) patients suffered from liver cirrhosis, whereby 116/142 (81.7%) were classified as Child-Pugh A, 23/142 (16.2%) as Child-Pugh B, and 3/142 (2.1%) as Child-Pugh C.

Median tumor size was 2.2 cm (1.0–8.5 cm) for 178 HCCs, and 3.0 cm (0.5–13 cm) for 371 metastases. A median of 2 (1–11) tumors were treated per ablation session (in total 273 sessions).

Table 1 demonstrates patient characteristics according to the performing radiologist. There was no significant difference between the groups.

### 2.3. SRFA Procedure

The method of SRFA has already been reported in detail elsewhere [11,12,13]. In brief, the whole procedure can be divided into five steps as follows:

1. Preparation: The entire procedure is carried out under general anesthesia with full muscle relaxation. Immobilization is provided by a single (Bluebag, Interventional Systems, Kitzbühel, Austria) or double vacuum fixation technique (BodyFix, Medical Intelligence, Schwabmünchen, Germany). For image-to-patient registration, 10–15 registration markers (Beekley Spots, Beekley Corporation, Bristol, CT, USA), are broadly attached to the skin.
2. Planning: A contrast-enhanced CT scan is acquired (Siemens SOMATOM Sensation Open, 82 cm bore size diameter, sliding gantry, Siemens AG, Erlangen, Germany) with 3 mm slice thickness in arterial and portal-venous phases. Datasets are transferred to an optical navigation system (Stealth Station Treon plus, Medtronic Inc., Boulder, CO, USA) and one or multiple antenna trajectories are planned with multiplanar and 3D reconstructed images using the navigation systems’ software.
3. Needle Placement: To compensate for respiratory motion, temporary disconnections of the endotracheal tube (ETT) are carried out during each CT scan and for needle placement. After registration and sterile draping, an ATLAS aiming device (Interventional Systems, Kitzbühel, Austria) is used for navigated trajectory alignment and the placement of 15G/17.2 cm coaxial needles (Bard Inc., Murray Hill, NJ, USA) without real-time imaging control, serving as guides and placeholders for the RF electrodes. After co-axial needle placement, a non-enhanced CT-scan is acquired to verify needle placement by image fusion with the planning CT scan using the navigation system’s image 3D registration algorithm.
4. RF Ablation: Up to three 17G RF probes (Cool-tip, Medtronic, Boulder, CO, USA, 3 cm exposure, 25 cm length) are inserted through the coaxial needles for serial tumor ablation, using the unipolar Cool-tip RF generator (Cool-tip, Medtronic, Boulder, CO, USA) and the Cool-tip RF switching controller for RF ablation. The standard ablation time for three RF probes is 16 min or until a significant increase in impedance (“roll-off effect”) is observed. Needle track ablation is performed prior to repositioning and final removal to reduce bleeding and potential tumor seeding.
5. Finalization: After ablation, a final contrast-enhanced CT scan is carried out in both arterial and portal venous phases for the assessment of complications and evaluation of the ablative safety margins in 3D. If needed, the intervention may be continued in the same session by additional placement of coaxial needles and subsequent ablation (e.g., residual tumor, lack of sufficient safety margin).

**Figure 2:**
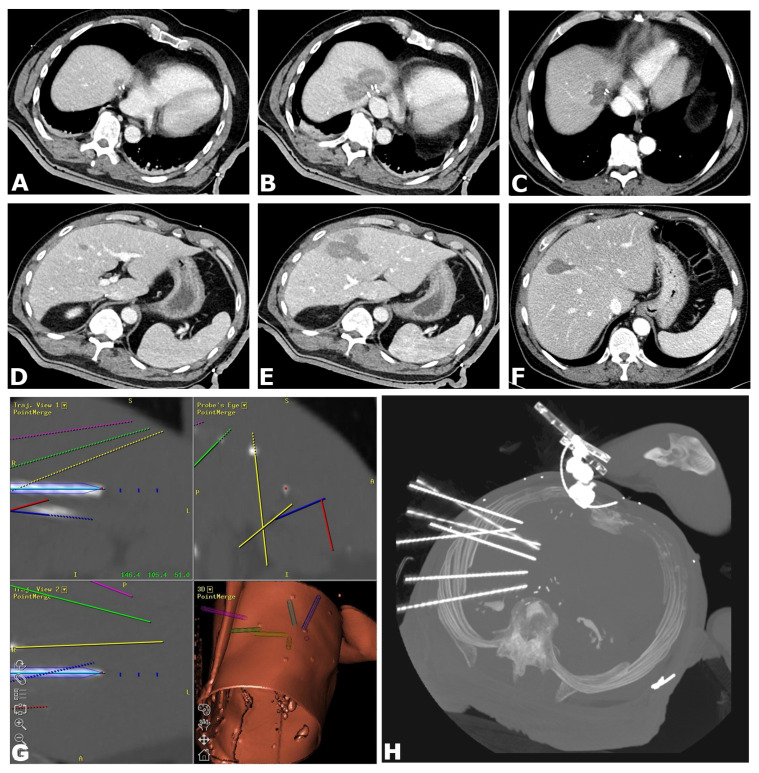
A 57-year old male with three colorectal cancer liver metastases (max 1.8 cm). (**A**) CT-image of the contrast-enhanced planning CT scan with a 1.8 cm recurrent metastasis following hepatic resection close to the central hepatic veins. (**B**) Immediate post-ablation and (**C**) 12-month contrast-enhanced CT scans showing no evidence of residual or recurrent disease. (**D**) Contrast-enhanced planning CT image showing a further 1.5 cm metastasis in segment V. (**E**) Immediate post-ablation and (**F**) 12-month CT scan showing no evidence of residual or recurrent disease. (**G**) Images from the navigation system depicting fused CT images of the needle control and planning scans with an example of a longitudinal planned trajectory (light blue) and the corresponding probe`s eye view (red dot) with perfect alignment. (**H**) maximum intensity projection image of the non-enhanced control CT scan showing all seven coaxial needles in position.

An example of an SRFA procedure is shown in Figure 2.

### 2.4. Endpoints

The endpoints of the present study were (a) technical success, (b) technical efficacy, (c) local recurrence, and (d) complication and mortality rates. 

These endpoints were defined as follows:

(a) Technical success: accurate coaxial needle placement, defined as less than 1cm maximum needle tip deviation according to predefined plans.

(b) Primary technical efficacy: complete ablation in a single SRFA session. 

(c) Secondary technical efficacy: complete ablation in more than one SRFA session, determined by follow-up imaging (either CT or MRI scans) at 1-month.

(d) Local recurrence: evidence of new enhancing nodules (CT or MRI scans at 3–6 months intervals) within or directly adjacent to the initially tumor-free ablation zone. 

(e) Complications were categorized according to the Society of Interventional Radiology (SIR) Standards of Practice Committee classification [14]. Mortality was defined as death within 30 days after SRFA treatment. 

To define endpoints, two board certified abdominal radiologists (with more than 10 years’ experience) evaluated the imaging results by consensus.

### 2.5. IR Experience

At baseline, the radiologist defined as a novice (IR 4) had already completed radiology training including basic CT- or US-guided procedures. However, IR4 did not have any experience with stereotactic procedures or thermal ablation. Prior to the start of the study, IR 4 attended five liver SRFA sessions in the previous four weeks. During the study period, procedural planning was executed by IR 4 alone but reviewed and altered, if necessary, by a more experienced IR. The novice IR (IR 4) performed all steps of the procedure including needle placement, RF probe positioning, RF ablation, RF probe extraction, and image fusion for ablation margin verification unsupervised, although an experienced IR was available in case of difficulties or questions. The more experienced IRs (IR 1–3) performed the complete procedure alone.

### 2.6. Procedural Time Measurements

The planning/placement time was calculated using the time difference between the time stamps of the planning CT scan and the non-contrast-enhanced control CT scan. This included time needed for planning, cleaning, sterile draping, and navigated coaxial needle placement. The total procedural time was calculated using the difference between the time stamps of the planning and final CT scans.

### 2.7. Statistical Analysis

Statistical analysis was performed using IBM SPSS version 27 (IBM, Armonk, NY, USA). Data were expressed as total numbers, median, and range. The differences between categorical variables were evaluated using the X^2^ test, and the differences between independent continuous variables were evaluated using the Mann-Whitney U or Kruskal Wallis Tests. A *p* value < 0.05 was considered as statistically significant.

## 3. Results

### 3.1. IR Experience

**Figure 3:**
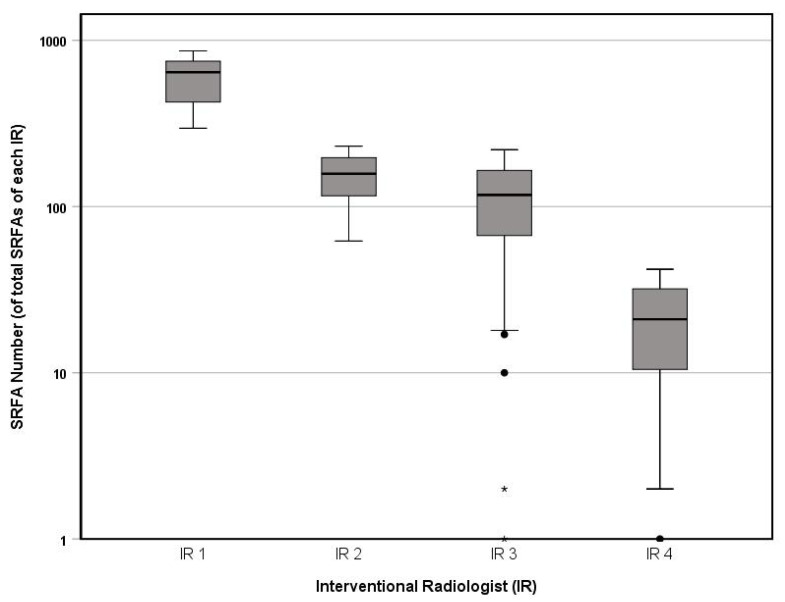
Boxplot comparison of continuous SRFA numbers based on the total executed SRFAs by each IR (logarithmic scale).

The median number of previously performed SRFAs at the time of procedures was 643 (range 296–864) for IR 1, 157.5 (62–231) for IR 2, 117.5 (1–220) for IR 3, and 21 (1–42) for IR 4, where differences between IR 4 and experienced IRs were statistically significant (*p* = 0.000). Figure 3 summarizes these results.

### 3.2. Procedural Time Efforts

The median planning/placement time was 76.5 min (range 32–271) for IR 1, 96 min (range 48–361) for IR 2, 95 min (range 43–231) for IR 3, and 119 min (range 46–292) for IR 4. Kruskal-Wallis Testing showed a significant difference in overall comparisons (*p* = 0.000). Overall median planning time for the experienced IRs was 92 min (range 32–361), being significantly less compared to IR 4, who had a median planning time of 119 min (*p* = 0.002).

Median total procedural time was 149.5 min (range 77–491) for IR 1, 204 min (range 95–454) for IR 2, 175 min (range 55–374) for IR 3, and 195 min (range 76–365) for IR 4. Kruskal-Wallis Testing showed a significant difference in overall comparisons (*p* = 0.000). The overall median total procedural time was 173 min (range 55–491) for experienced IRs, demonstrating no statistically significant differences compared to IR 4 with 195 min (*p* = 0.059).

**Figure 4:**
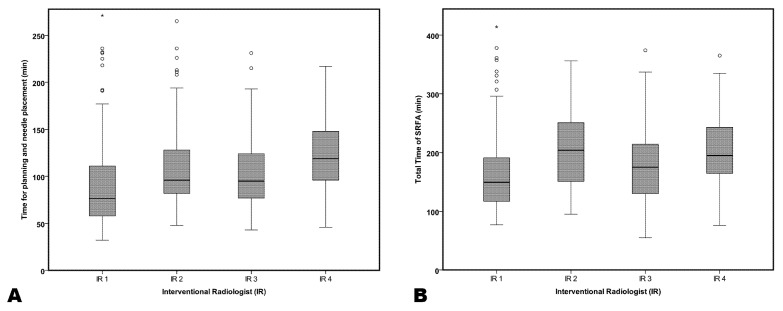
Boxplot comparison of time needed for (**A**) planning/placement and (**B**) the whole procedure grouped by interventional radiologists (IR). IR 1 reflects the most and IR 4 the least experienced operator.

Figure 4 shows box plots of the presented results.

### 3.3. Safety

Major complications occurred in 18 of 273 ablations (6.6%), with two deaths due to major hemorrhage with subsequent hemorrhagic shock, and one death due to systemic spread of local infection leading to septic shock (mortality rate 1.1% [3/273]). Thermal injury to the diaphragm and access route led to pleuro-cutaneous fistulation in one case, which required repeated thoracenteses. Two temporary episodes of respiratory failure required intensive care admission. Three liver abscesses were treated by percutaneous drainage. Three pneumothoraces and four instances of perihepatic hemorrhage were successfully treated by the IR in the same anesthetic session by placement of thoracocostomy tubes/transarterial embolization and did not affect the postoperative course. Two pleural effusions required thoracentesis.

The major complication and mortality rate was 6.8% (16/234 ablations) and 1.3% (3/234) for experienced IRs compared to 5.1% (2/39) and 0% (0/39) for the novice without significant differences (*p* = 0.477 and *p* = 0.690, respectively).

The median hospital stay after ablation was 4 days (1–31) for experienced IRs and 5 days (2–24) for the novice IR without a significant difference (*p* = 0.852).

Per ablation session, three RF probes were simultaneously advanced through 1–28 (median 4) coaxial needles.

### 3.4. Technical Success and Local Tumor Control

Coaxial needles were inserted according to plan by all IRs in all 628 tumors (technical success rate 100%). The overall primary technical efficacy rate was 98.6% (619/628) and the secondary technical efficacy rate was 99.5% (625/628).

Both primary technical efficacy rate (98.5% (525/533) vs. 98.9% (94/65); *p* = 0.735) and secondary technical efficacy rate (99.6% (531/533) vs. 98.9% (94/65); *p* = 0.378) revealed no significant difference when comparing experienced IRs vs. novice IR.

The overall local recurrence rate (LR) was 5.6% (35 of 628 tumors) after median imaging follow-up of 9.3 months (range 1–74 months). Local recurrence rates were almost identical (*p* = 0.886) for experienced IRs vs. novice IR with 5.6% (30/533) and 5.3% (5/95), respectively.

Details of the results are presented in Table 2 and Table 3.

## 4. Discussion

This study demonstrates that stereotactic radiofrequency ablation (SRFA) can be performed by an interventional radiologist (IR) with little experience, achieving similar results to those of more experienced IRs in terms of safety, technical success, and local tumor control. Thus, it can be assumed that that SRFA represents a highly reproducible technique that can easily be learned by any radiologist, even with little experience in interventional procedures. The reason for this is most likely the reliability of the navigated ablation device used in this technique.

The technical success was 100% for all IRs coupled with a primary technical efficacy rate between 97.2–100%, despite inclusion of large, irregularly shaped tumors with up to 15 cm and the necessity of using up to 28 coaxial needles. This finding differs considerably from the previous literature on conventional US-guided ablation techniques, considering Poon et al. [8] reported significantly lower complete ablation rates in untrained operators (84% vs. 100%) and Hildebrand et al. [6] showed a higher complete ablation rate in the more experienced group (93.7% vs. 96.2%) using ultrasound as imaging modality.

In order to ascend the learning curve in ultrasound guided ablations, it is essential to gain competency in both diagnostic and interventional ultrasound. For example, Takai Takamatsu et al. [15] proposed a training program for US-guided RFA including weekly diagnostic US sessions as a cornerstone. However, it occurs very commonly in US-guided interventions that interventions tend to be extremely difficult or impossible to execute for non-experts due to unfavorable target locations. In contrast to this, stereotaxy offers three-dimensional ablation path planning where difficulties regarding the access routes or the target tumor, such as lesions in the hepatic dome [16] or within the caudate lobe [17], can be overcome. Furthermore, this method enables optimal alignment of multiple RF probes with overlapping ablation zones in order to reliably extend the spectrum of percutaneous tumor ablation to large, irregularly shaped or multiple target tumors. One of the key aspects of this method is the usage of an aiming device. This device ensures very accurate path alignment and targeting even in untrained hands.

The time required for planning and needle placement was significantly higher for the IR novice, which is most likely related to the fact that planning in three-dimensional space has a learning curve, and additional time is needed when checking the novice’s plans. However, time differences for entire procedures were much less pronounced (statistically slightly not significant, *p* = 0.059), confirming once more the steep learning curve with regard to practical execution.

Another factor in the steep learning curve of SRFA is that important workflows, such as virtual planning of trajectories or aiming device adjustment, can be practiced and learned ex-vivo, i.e., without patients (in contrast to US-guided interventions). Furthermore, in view of increasing global technical networking, the already existing possibility of remote access to the planning station could gain an increasing significance. For example, it would be possible to control or even execute planning remotely.

The overall local recurrence rate was 5.6% in the present study and varied from 3.8–10.1% across operators (*p* = 0.051). This trend can be attributed to higher local recurrence rates seen in the second most experienced IR with 10.1%, since other rates ranged between 3.8% and 5.3%. This effect could have been related to the complexity of the cases. In contradistinction, Lee et al. [7] showed a significant inverse correlation between operator experience and tumor recurrence rates in a study of 2827 patients undergoing RFA for HCC. Similarly, Hildebrand et al. [6] reported higher local recurrence rates in less experienced operators at 15.9% vs. 9.5% for the more experienced group. However, Takai Takamatsu et al. [15] reported similar local recurrence rates after RFA for HCC in the trainee and the mentor group with 8.8% vs. 7.7%, respectively, in a RFA training program with supervision. 

The overall major complication rate was 6.6% and did not significantly differ between operators (*p* = 0.498), ranging between 5.1% and 10.3%. Interestingly, the most experienced IR had the highest complication rate, which is likely attributable to a higher case complexity. The major complication rate is similar to the published literature following percutaneous liver tumor ablation at 0.9–10.0% [1,18,19]. However, the lack of differences between operators in this study is in contrast to the findings of similar studies. In fact, Lee et al. [7] found a significantly higher red blood cell transfusion rate and Poon et al. [8] reported significantly higher morbidity rates (16% vs. 4%) for interventions performed by less experienced operators. In addition, despite the multiprobe approach, the overall complication rates in the presented study are lower than those reported in the comparable laparoscopic RFA literature at 10–12.4% [20,21].

We are well aware of the limitations of the presented study, including the retrospective design, heterogeneity of treatments in terms of difficulty, and a single treatment center bias. Furthermore, all plans from the novice IR were checked before needle placement, which could have affected the outcome. Comparison with previous similar studies is also limited because there has been little adoption of stereotactic navigation systems until recently.

## 5. Conclusions

SRFA is a safe, effective, and reliable treatment option for malignant liver tumors, and favorable outcomes can be achieved even by inexperienced operators with minimal supervision.

## Figures and Tables

**Table 1:** Patient characteristics of 237 patients undergoing 273 SRFAs of primary and metastatic liver tumors, grouped by interventional radiologist.

Patient Characteristics	All	IR 1	IR 2	IR 3	IR 4	*p*-Value
Ablations, n	273	78	78	78	39	0.924
HCC, n (%)	170 (62.3)	45 (57.7)	48 (61.5)	53 (67.9)	24 (61.5)	
ICC, n (%)	9 (3.3)	3 (3.8)	3 (3.8)	2 (2.6)	1 (2.6)	
Mets, n (%)	94 (34.3)	30 (38.5)	27 (34.6)	23 (29.5)	14 (35.9)	
Age, median years (range)	69 (18–88)	70 (38–84)	71 (38–88)	69 (44–84)	66 (18–80)	0.117
Sex (female/male), n (%)	91/182 (33/67)	32/46 (41/59)	22/56 (28/72)	24/54 (31/69)	13/26 (33/67)	0.357
Cirrhosis, n (%)	162/273 (59.3)	43/78 (55.1)	45/78 (57.7)	50/78 (64.1)	25/39 (64.1)	0.701
Child A, n (%)	135/162 (83.3)	38/43 (88.4)	33/45 (73.3)	41/50 (82.0)	24/25 (61.5)	
Child B, n (%)	24/162 (14.8)	5/43 (11.6)	11/45 (24.4)	7/50 (14.0)	1/25 (4.0)	
Child C, n (%)	3/162 (1.9)	-	1/45 (2.2)	2/50 (4.0)	-	
Max. Tumor Size, median (range)	2.8 cm (0.5–15)	3.0 cm (0.5–15)	3.0 cm (0.5–10)	2.4 cm (0.5–13)	2.5 cm (0.9–13.5)	0.373
HCC, n (%)	2.6 cm (0.8–15)	3.0 cm (0.5–15)	2.9 cm (1–8.3)	2.3 cm (1.1–9.0)	2.4 cm (0.9–6.3)	
ICC, n (%)	5.5 cm (0.5–8.0)	2.0 cm (0.5–5.5)	5.4 cm (2.8–8.0)	7.0 cm (6.0–8.0)	6.7 cm (6.7–6.7)	
Mets, n (%)	2.7 cm (0.8–13.5)	3.0 cm (0.8–13.5)	3.5 cm (0.8–10.0)	2.4 cm (1.0–13.0)	3.0 cm (1.1–13.5)	
Tumor Number, n (range)	2 (1–11)	1 (1–11)	2 (1–6)	2 (1–11)	1 (1–8)	0.133
HCC, n (%)	2 (1–9)	2 (1–9)	1 (1–4)	2 (1–6)	2 (1–8)	
ICC, n (%)	2 (1–3)	2 (1–3)	1.5 (1–2)	1.5 (1–2)	1 (1–1)	
Mets, n (%)	1 (1–11)	1 (1–11)	2 (1–6)	3 (1–11)	1 (1–5)	
Needles, n (range)	4 (1–28)	4 (1–28)	5 (1–12)	4 (1–14)	5 (1–20)	0.159
HCC, n (%)	4 (1–20)	4 (1–20)	5 (1–12)	4 (1–12)	4 (1–20)	
ICC, n (%)	6 (1–11)	4 (4–7)	8 (6–11)	4 (1–7)	10 (10–10)	
Mets, n (%)	4 (1–28)	5 (1–28)	5 (1–12)	4 (2–14)	6 (3–19)	

**SRFA** = stereotactic radiofrequency ablation, **HCC** = hepatocellular carcinoma, **ICC** = intrahepatic cholangiocarcinoma, **METS** = metastatic liver tumors, **Child** = Child Pugh Score.

**Table 2:** Ablation Results grouped by IR.

	Overall	IR 1	IR 2	IR 3	IR 4	*p*-Value
Mortality rate, n (%)	3/273 (1.1)	1/78 (1.3)	1/78 (1.3)	1/78 (1.3)	0/39 (0)	0.918
Major Complication Rate, n (%)	18/273 (6.6)	8/78 (10.3)	4/78 (5.1)	4/78 (5.1)	2/39 (5.1)	0.498
HCC, n (%)	12/170 (7.1)	6/45 (13.3)	1/48 (2.1)	3/53 (5.7)	2/22 (8.3)	0.193
ICC, n (%)	2/9 (22.2)	0/3 (0)	2/3 (66.7)	0/2 (0)	0/1 (0)	0.162
METS, n (%)	4/94 (4.3)	2/30 (6.7)	1/27 (3.7)	1/23 (4.3)	0/14 (0)	0.944
Hospital Days, median (range)	5 (1–31)	4 (1–24)	5 (2–21)	4 (1–31)	5 (2–24)	0.257
HCC, n (%)	4 (1–20)	4 (1–20)	5 (3–14)	5 (1–18)	5 (2–10)	0.195
ICC, n (%)	6 (2–19)	3 (2–7)	12 (5–19)	4.5 (3–6)	7 (7–7)	0.206
METS, n (%)	5 (1–31)	6 (2–24)	5 (2–21)	4 (1–31)	6 (2–24)	0.944
Technical Success, n (%)	628/628 (100)	176/176 (100)	149/149 (100)	208/208 (100)	95/95 (100)	-
Primary Technical Efficacy, n (%)	619/628 (98.6)	171/176 (97.2)	149/149 (100)	205/208 (98.6)	94/95 (98.9)	0.192
HCC, n (%)	355/363 (98.8)	98/102 (96.1)	81/81 (100)	111/114 (97.4)	65/66 (98.5)	0.325
ICC, n (%)	14/14 (100)	6/6 (100)	4/4 (100)	3/3 (100)	1/1 (100)	-
METS, n (%)	250/251 (99.6)	67/68 (98.5)	64/64 (100)	91/91 (100)	28/28 (100)	0.440
Secondary Technical Efficacy, n (%)	625/628 (99.5)	176/176 (100)	149/149 (100)	206/208 (99.0)	94/95 (98.9)	0.355
HCC, n (%)	360/363 (99.2)	102/102 (100)	81/81 (100)	112/114 (98.2)	65/66 (98.5)	0.376
ICC, n (%)	14/14 (100)	6/6 (100)	4/4 (100)	3/3 (100)	1/1 (100)	-
METS, n (%)	251/251 (100)	68/68 (100)	64/64 (100)	91/91 (100)	28/28 (100)	-
Local Recurrence, n (%)	35/628 (5.6)	7/176 (4.0)	15/149 (10.1)	5/95 (5.3)	5/95 (5.3)	0.051
HCC, n (%)	16/363 (4.4)	2/102 (2.0)	7/81 (8.6)	5/114 (4.5)	2/66 (3.0)	0.158
ICC, n (%)	1/14 (7.1)	0/6 (0)	0/4 (0)	1/3 (33.3)	0/1 (0)	0.267
METS, n (%)	18/251 (7.2)	5/68 (7.4)	8/64 (12.5)	2/91 (2.2)	3/28 (10.7)	0.084
Time Efforts						
Planning and Placement, min (range)	76.5 (32–271)	96.0 (48–361)	76.5 (32–271)	76.5 (32–271)	119 (46–292)	0.000
Total Time, min (range)	149.5 (77–491)	204 (95–454)	95.0 (43–231)	149.5 (77–491)	195 (76–365)	0.000

**SRFA** = stereotactic radiofrequency ablation, **HCC** = hepatocellular carcinoma, **ICC** = cholangiocellular carcinoma, **METS** = metastatic liver tumors, **IR** = interventional radiologist.

**Table 3:** Ablation Results grouped by experienced IRs (IR1–IR3) and novice IR (IR 4).

	Experienced IRs	IR 4	*p*-Value
Mortality rate, n (%)	3/234 (1.3)	0/39 (0)	0.477
Major Complication Rate, n (%)	16/234 (6.8)	2/39 (5.1)	0.690
HCC, n (%)	10/146 (6.8)	2/24 (8.3)	0.793
ICC, n (%)	2/8 (25.0)	0/1 (0)	0.571
METS, n (%)	4/80 (5.0)	0/14 (0)	0.393
Hospital Days, median (range)	4 (1–31)	5 (2–24)	0.852
HCC, n (%)	4 (1–20)	5 (2–10)	0.977
ICC, n (%)	5.5 (2–19)	7 (7–7)	0.667
METS, n (%)	5 (1–31)	6 (2–24)	0.873
Technical Success, n (%)	533/533 (100)	95/95 (100)	-
Primary Technical Efficacy, n (%)	525/533 (98.5)	94/95 (98.9)	0.735
HCC, n (%)	290/297 (97.6)	65/66 (98.5)	0.674
ICC, n (%)	13/13 (100)	1/1 (100)	-
METS, n (%)	222/223 (99.6)	28/28 (100)	0.674
Secondary Technical Efficacy, n (%)	531/533 (99.6)	94/95 (98.9)	0.378
HCC, n (%)	360/297 (99.2)	65/66 (98.5)	0.494
ICC, n (%)	13/13 (100)	1/1 (100)	-
METS, n (%)	223/223 (100)	28/28 (100)	-
Local Recurrence, n (%)	30/533 (5.6)	5/95 (5.3)	0.886
HCC, n (%)	14/297 (4.7)	2/66 (3.0)	0.547
ICC, n (%)	1/13 (7.7)	0/1 (0)	0.773
METS, n (%)	15/223 (6.7)	3/28 (10.7)	0.441
Time Efforts			
Planning and Placement, min (range)	92 (32–361)	119 (46–292)	0.002
Total Time, min (range)	173 (55–491)	195 (76–365)	0.059

**SRFA** = stereotactic radiofrequency ablation, **HCC** = hepatocellular carcinoma, **ICC** = cholangiocellular carcinoma, **METS** = metastatic liver tumors, **IR** = interventional radiologist.

## Data Availability

The data presented in this study are available on request from the corresponding author.
